# Presence of Late Gadolinium Enhancement by Cardiac Magnetic Resonance Among Patients With Suspected Cardiac Sarcoidosis Is Associated With Adverse Cardiovascular Prognosis: A Systematic Review and Meta-Analysis

**DOI:** 10.1161/CIRCIMAGING.116.005001

**Published:** 2016-09

**Authors:** Edward Hulten, Vikram Agarwal, Michael Cahill, Geoff Cole, Tomas Vita, Scott Parrish, Marcio Sommer Bittencourt, Venkatesh L. Murthy, Raymond Kwong, Marcelo F. Di Carli, Ron Blankstein

**Affiliations:** Non-Invasive Cardiovascular Imaging Program, Departments of Medicine and Radiology, Brigham and Women's Hospital and Harvard Medical School, Boston, MA (E.H., V.A., T.V., R.K., M.F.D.C., R.B.); Cardiology Service (E.H., M.C., G.C.) and Pulmonology Service (S.P.), Division of Medicine, Walter Reed National Military Medical Center and Uniformed Services University of Health Sciences, Bethesda, MD; Center for Clinical and Epidemiological Research, University Hospital and São Paulo State Cancer Institute–University of São Paulo, Brazil (M.S.B.); Preventive Medicine Center–Hospital Israelita Albert Einstein, São Paulo, Brazil (M.S.B); Frankel Cardiovascular Center, Department of Internal Medicine, Division of Cardiovascular Medicine and Department of Radiology, Division of Nuclear Medicine and Cardiothoracic Imaging, University of Michigan, Ann Arbor (V.L.M.)

**Keywords:** cardiac arrhythmia, cardiomyopathies, heart conduction system, magnetic resonance imaging, sarcoidosis

## Abstract

**Background:**

Individuals with cardiac sarcoidosis have an increased risk of ventricular arrhythmia and death. Several small cohort studies have evaluated the ability of late gadolinium enhancement (LGE) by cardiac magnetic resonance imaging (MRI) to predict adverse cardiovascular events. However, studies have yielded inconsistent results, and some analyses were underpowered. Therefore, we sought to systematically review and perform meta-analysis of the prognostic value of cardiac MRI for patients with known or suspected cardiac sarcoidosis.

**Methods and Results:**

We systematically searched for cohort studies of patients with known sarcoidosis with suspected cardiac involvement who underwent cardiac MRI with LGE with at least 12 months of either prospective or retrospective follow-up data regarding post-MRI adverse cardiovascular outcomes. We identified 7 studies of 694 subjects (mean age 53; 42% men). One hundred and ninety-nine patients (29%) were LGE positive. All-cause mortality occurred in 19 LGE-positive versus 17 LGE-negative subjects (annualized incidence, 3.1% versus 0.6%). The pooled relative risk was 3.38 (95% confidence interval, 1.07-10.7; *P*=0.04). Cardiovascular mortality occurred in 10 LGE-positive versus 2 LGE-negative subjects (annualized incidence, 1.9% versus 0.3%; relative risk 10.7 [95% confidence interval, 1.34–86.3]; *P*=0.03). Ventricular arrhythmia occurred in 41 LGE-positive versus 0 LGE-negative subjects (annualized incidence, 5.9% versus 0%; relative risk 19.5 [95% confidence interval, 2.68–143]; *P*=0.003). A combined end point of death or ventricular arrhythmia occurred in 64 LGE-positive versus 18 LGE-negative subjects (annualized incidence, 8.8% versus 0.6%; relative risk 6.20 [95% confidence interval, 2.47–15.6]; *P*<0.001). There was no significant heterogeneity for any outcomes.

**Conclusions:**

LGE is associated with future cardiovascular death and ventricular arrhythmia among patients referred to MRI for known or suspected cardiac sarcoidosis.

Cardiac sarcoidosis (CS) is an increasingly recognized infiltrative heart disease that can cause heart failure, left or right ventricular systolic dysfunction, atrioventricular block, arrhythmias, and sudden cardiac death.^1^ The diagnosis of CS often relies on cardiac imaging, as clinical criteria and endomyocardial biopsy both have limited diagnostic accuracy.^1,2^ However, even with the use of advanced imaging techniques such as cardiac magnetic resonance (CMR) imaging and cardiac positron emission tomography (PET), the diagnosis may not always be clear, and most studies assessing the diagnostic accuracy of these techniques have been limited by the lack of a reliable reference standard.^3^ As a result, recent studies have focused on evaluating the prognostic value of various cardiac imaging findings, as the risk of future events is essential for deciding on the role of therapies that are often toxic and costly.

Several CMR studies have evaluated patients with suspected CS and have shown that the presence of late gadolinium enhancement (LGE) was associated with a higher rate of adverse events. Yet, many of these studies were small and relied on various composite end points because of limited statistical power. Moreover, several studies yielded inconsistent results. For example, 2 studies concluded that LGE was associated with all-cause mortality,^4,5^ whereas 2^6,7^ demonstrated a nonsignificant trend toward increased risk, and 3 other studies did not identify any increased risk of LGE for all-cause mortality.^8–10^ In fact, 1 study did not find an association between LGE and any adverse events.^10^ As a result, the magnitude of the association between LGE and adverse events has been unclear.

Given the increasing role of CMR in evaluating patients with suspected CS, we sought to perform a systematic review and meta-analysis of all studies evaluating the association of CMR findings with downstream cardiac death and ventricular arrhythmias, hard end points that are highly relevant for clinical decision making. Furthermore, given the high event rates that have been observed in patients with suspected sarcoidosis,^11,12^ we were also interested in identifying the value of a negative CMR to identify low-risk individuals.

## Methods

### Literature Search

We followed the statement for Preferred Reporting in Systematic Review and Meta-Analysis.^13^ The Preferred Reporting in Systematic Review and Meta-Analysis checklist is provided in Table I in the Data Supplement. The study protocol has not been previously published. We used a systematic search strategy of EMBASE, Pubmed, and Web of Science to identify prospective or retrospective cohort studies of at least 20 patients with suspected or confirmed CS who were referred for cardiac magnetic resonance imaging (MRI) with the evaluation of LGE and at least 12 months of clinical follow-up after MRI for any of the following end points: all-cause mortality, cardiovascular mortality, ventricular arrhythmia, heart block requiring pacemaker implantation, or heart failure requiring hospital admission. We used the search terms and corresponding MeSH headings cardiac sarcoidosis MRI from index date to January 26^th^, 2016. The full search terms are included in the Methods in the Data Supplement. We additionally searched references of articles reviewed. We did not restrict by language. There was no external funding source, and all data analyzed were abstracted from the published article without unpublished or individual patient level data.

We excluded studies that did not report clinical outcomes. We furthermore excluded studies that evaluated patients only by nuclear imaging or echocardiography, that did not report clinical outcomes but were purely diagnostic accuracy studies, or that did not classify patients as having LGE by cardiac MRI.

### Data Abstraction

Two authors (E.H. and V.A.), working independently, abstracted raw data onto a standardized form that recorded study characteristics (design, demographics, sample size, and inclusion and exclusion criteria), MRI findings including LGE positivity and quantification, and included clinical outcomes stated above.

### Data Synthesis

For the primary analysis, we evaluated all-cause mortality according to LGE positivity. Secondary analyses included cardiovascular mortality, ventricular arrhythmia (appropriate implantable cardioverter defibrillator [ICD] shock of ventricular arrhythmia excluding anti-tachycardia pacing; ventricular fibrillation, or sustained ventricular tachycardia >30 seconds), and combined death or ventricular arrhythmia. We also abstracted bradyarrhythmia resulting in pacemaker implantation and hospital admission for heart failure. We generated 2×2 tables from the data reported in each article and pooled the outcomes using a random effects model to calculate summary of annualized incidences for each outcome according to LGE positivity in addition to the pooled relative risk (RR) for each adverse clinical outcome according to LGE positivity.

### Sensitivity Analysis

We examined heterogeneity using visual inspection via Galbraith plots in addition to statistical analysis by Q and I^2^ statistics.^14^ The I^2^ statistics provide an estimate of the variance because of heterogeneity rather than chance alone and is based on the traditional measure of variance, the Cochrane Q statistic. We assessed for small study effects with linear regression for binary outcomes by Harbord test.^15^

### Quality Assessment

Two authors (M.C. and G.C.) working independently assessed the quality using the Newcastle–Ottawa scale, which is used for assessing the quality of nonrandomized studies in meta-analysis, as recommended by the Cochrane collaboration.^16^ This scale rates quality from 0 to 9 based on 8 questions involving patient selection, comparability, and outcomes assessment. Disagreements were resolved by consensus.

### Statistical Analysis

All analyses were conducted using Stata version 12.1 (StataCorp, College Station, TX), with the *metan* commands. *P* values were 2 sided with an alpha of 0.05 considered statistically significant.

## Results

**Figure 1:**
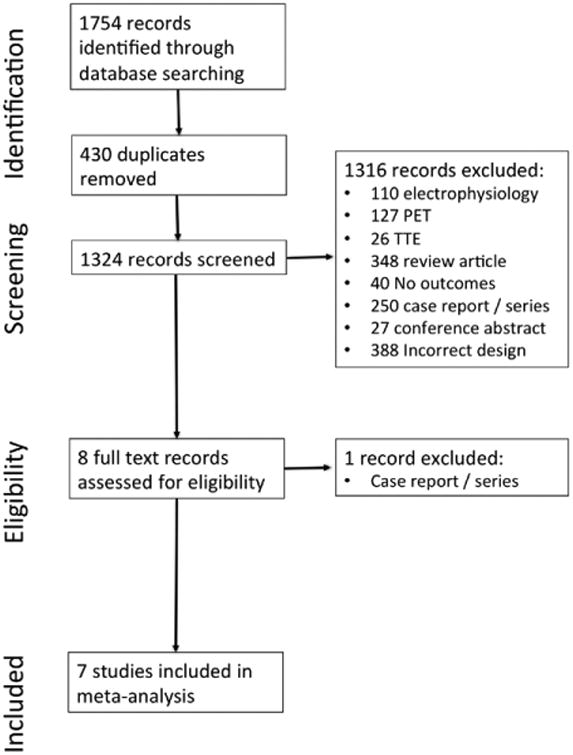
Literature search results. PET indicates positron emission tomography.

The Preferred Reporting in Systematic Review and Meta-Analysis flow chart for literature search is presented in Figure 1. Seven observational studies of 694 patients were included.^4–10^ 681 patients (98%) had biopsy-proven or met radiological criteria for diagnosis of extra-CS, whereas 13 patients were suspected on presentation of having isolated CS. The mean age of the population was 53±4 years. There were 289 (42%) men. Demographics for the study population are further presented in Table 1. 199 patients (29%) were LGE positive. Similar patients were enrolled in each study (patients with extra-CS and clinical referral because of symptoms of suspected cardiac involvement of sarcoidosis) although Nagai et al^10^ evaluated an entirely asymptomatic screening population. Similar methods for acquisition and interpretation of LGE were reported, except that Nagai et al^10^ reported the use of a balanced steady state free precession (as opposed to gradient recall echo in all other studies) with fixed inversion time of 300 ms in contrast to other studies that reported adjusting inversion time consistent with usual practice. Gadolinium dose reported ranged from 0.1 to 0.20 mmol/kg consistent with usual practice (Table 2).Reporting of ventricular arrhythmia used similar methods in each study to target clinically significant ventricular tachycardia, ventricular fibrillation, ICD shock, or sudden cardiac death. Two articles reported nonsustained ventricular tachycardia, which we separated from our outcomes (Table 3).

### All-Cause Mortality

Among 6 studies reporting all-cause mortality, there were 19 deaths among subjects with LGE and 17 deaths among subjects without LGE (Table 4).

**Figure 2:**
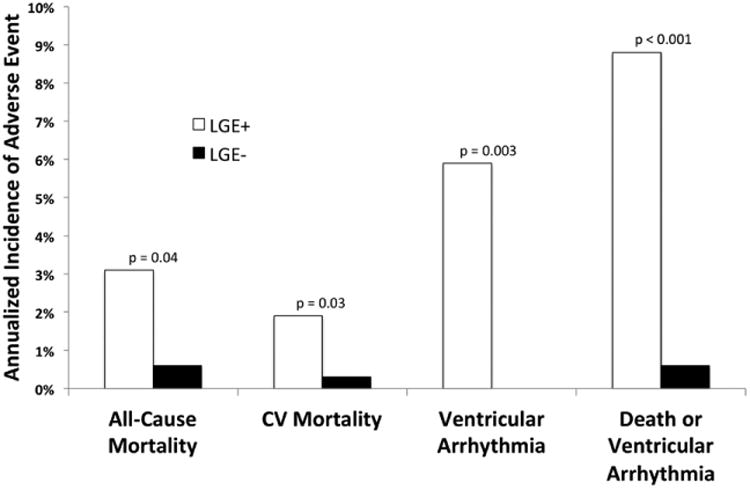
Annualized event rates according to late gadolinium enhancement (LGE) positive vs negative. CV indicates cardiovascular.

**Figure 3:**
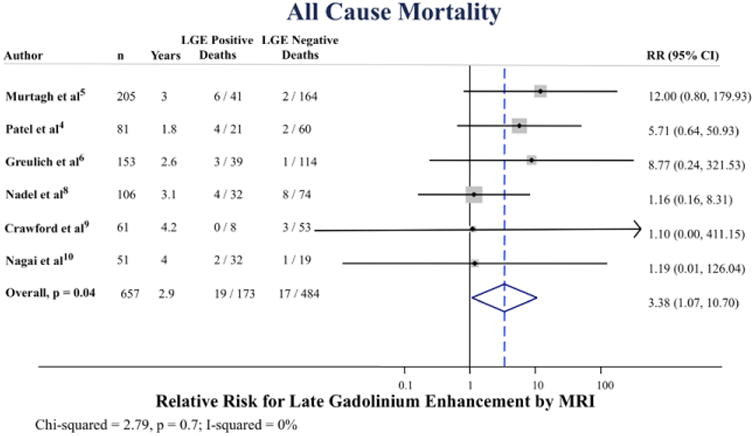
Pooled relative risk (RR) for all-cause mortality according to late gadolinium enhancement (LGE) positive vs negative. CI indicates confidence interval and MRI, magnetic resonance imaging.

The annualized incidence of mortality was 3.1% versus 0.6% for LGE positive versus LGE negative, respectively (Figure 2). Three studies demonstrated an increased risk of all-cause mortality associated with LGE and 3 did not. The pooled RR was 3.38 ([95% confidence interval, 1.07–10.7]; *P*=0.04), Figure 3. There was no statistical evidence of heterogeneity (I^2^=0%; *P*=0.7).

### Cardiovascular Mortality

**Figure 4:**
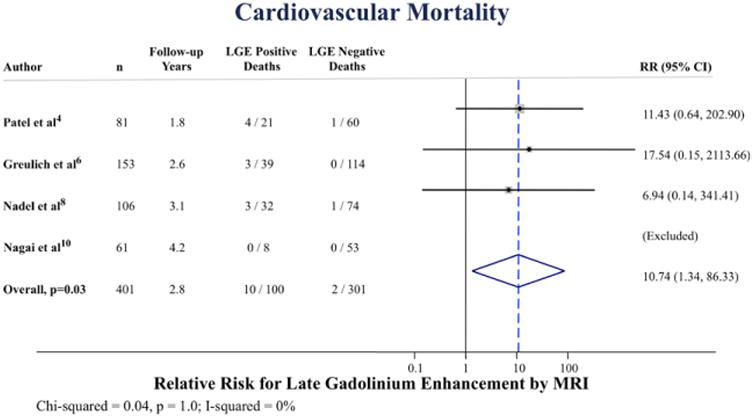
Pooled relative risk (RR) for cardiovascular mortality according to late gadolinium enhancement (LGE) positive vs negative. CI indicates confidence interval and MRI, magnetic resonance imaging.

Among 4 studies reporting cardiovascular mortality, there were 10 deaths among subjects with LGE and 2 deaths among subjects without LGE. The annualized incidence of cardiovascular mortality was 1.9% versus 0.3% for LGE positive versus LGE negative, respectively (Figure 2). The pooled RR was 10.7 ([95% confidence interval, 1. 34–86.3]; *P*=0.03), Figure 4. There was no statistical evidence of heterogeneity (I^2^=0%; *P*=1.0).

### Ventricular Arrhythmia

**Figure 5:**
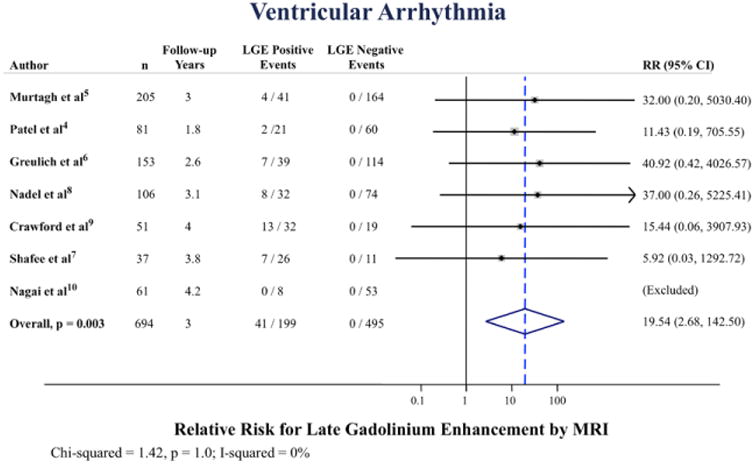
Pooled relative risk (RR) for ventricular arrhythmia according to late gadolinium enhancement (LGE) positive vs negative. CI indicates confidence interval and MRI, magnetic resonance imaging.

Among 7 studies reporting ventricular arrhythmia (appropriate ICD therapy of ventricular arrhythmia, ventricular fibrillation, or sustained ventricular tachycardia >30 seconds), there were 41 occurrences among subjects with LGE and none among subjects without LGE. The annualized incidence of ventricular arrhythmia was 5.9% versus 0% for LGE positive versus LGE negative, respectively (Figure 2). The pooled RR was 19.5 ([95% confidence interval, 2.68–143]; *P*=0.003), Figure 5. There was no statistical evidence of heterogeneity (I^2^=0%; *P*=1.0).

### Death or Ventricular Arrhythmia

**Figure 6:**
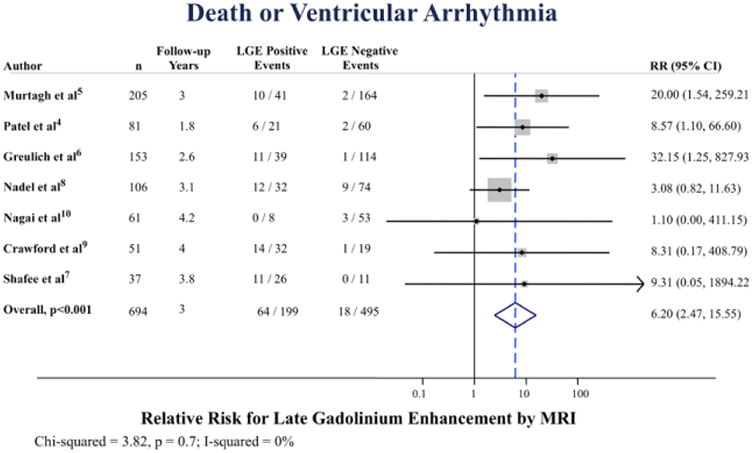
Pooled relative risk (RR) for combined death or ventricular arrhythmia according to late gadolinium enhancement (LGE) positive vs negative. CI indicates confidence interval and MRI, magnetic resonance imaging.

Among 7 studies reporting a combined end point of death or ventricular arrhythmia, there were 64 occurrences among subjects with LGE and 18 among subjects without LGE. The annualized incidence of death or ventricular arrhythmia was 8.8% versus 0.6% for LGE positive versus LGE negative, respectively (Figure 2). The pooled RR was 6.20 ([95% confidence interval, 2.47–15.6]; *P*<0.001), Figure 6. All studies demonstrated a trend toward or statistically significantly increased risk for those with LGE, except for Nagai et al^10^, which was a screening cohort with a low prevalence of corticosteroid use, a low prevalence of disease (13% LGE+) and without any cardiac-specific adverse events. There was no statistical evidence of heterogeneity (I^2^=0%; *P*=0.7).

### Outcomes With Insufficient Data for Analysis

Our a priori methods called for analysis of the outcomes of heart block requiring pacemaker implantation and hospital admission for heart failure. However, only 3 studies reported the outcome of heart block requiring pacemaker implantation and 2 reported heart failure outcomes. These end points were summarized (Tables 1 and 4) qualitatively without meta-analysis. Additionally, our methods sought to abstract detailed information regarding baseline corticosteroid or immunotherapy use. Although all studies reported qualitative information regarding baseline corticosteroid and immunotherapy use, most studies did not distinguish between current or prior use. Additionally, corticosteroid dosage and duration and adjunctive immunotherapy were heterogeneous (Table 1). Therefore, a summary of reported immunotherapy is provided with no statistical analysis.

### Quality Assessment and Evaluation for Small Study Effects

Studies were rated as high quality with a median of 8 points by the Newcastle Ottawa Scale. Cases and controls were drawn from similarly referred populations in each study, with clear definition of exposure (LGE) and outcomes. All studies had complete or nearly complete follow-up. Three studies did not make attempts to adjust, although this was related to the lack of statistical power. The median quality score was 8 (range, 7–9; Table II in the Data Supplement). There was no evidence for small study effects for any of the outcomes (*P*=0.9 for mortality; *P*=0 0.8 for cardiovascular mortality; *P*=0.9 for death or ventricular arrhythmia; and *P*=0.1 for ventricular arrhythmia).

## Discussion

This systematic review and meta-analysis of LGE prognosis among patients with known or suspected CS offers comprehensive and robust data that CMR is useful in stratifying patients into low or high risk for future cardiac death and ventricular arrhythmias. Although several prior studies evaluated the prognostic utility of CMR in patients with suspected CS, these studies have been limited by small sample sizes and low event rates, which have resulted in a large variability in effect size. In our analysis of 7 observational studies, which included 694 patients, with a mean follow-up duration of 36 months, we found that patients with LGE on CMR had 10.8% annualized incidence of either death or ventricular arrhythmias versus 0.6% for those without LGE (relative risk, 5.5; *P*<0.001). Furthermore, the event rates among patients without LGE on CMR as compared with patients with LGE on CMR were consistently lower and statistically significant for all outcomes, including all-cause mortality, cardiac death, and ventricular arrhythmias.

Overall, CS carries a high risk of death and ventricular arrhythmia, thus a diagnosis of CS bears significant therapeutic and prognostic implications. Clinical guidelines have been proposed by the Japanese Ministry of Health and Welfare criteria to aid in the diagnosis of CS. These guidelines were initially described in 1993, ^17^ and subsequently updated in 2007.^18^ However, these criteria have not been systematically validated and have lower sensitivity and specificity when compared with imaging modalities including CMR^3,19^ and 2-deoxy-2-[fluorine-18] fluoro-deoxyglucose positron emission tomography (^18^F-FDG PET) imaging.^20^ It is worth noting that the 2007 update of the Japanese Ministry of Health and Welfare criteria has included the presence of LGE on CMR as one of the minor criterion to aid in the diagnosis of CS. Recently, the Heart Rhythm Society also introduced an expert consensus statement, which has also not been validated scientifically but does acknowledge the role of LGE on CMR to aid in the diagnosis of CS in patients with syncope or presyncope, abnormal ECG, and abnormal echocardiogram. These Heart Rhythm Society experts recommend consideration of the presence of LGE for referral to diagnostic electrophysiological study to evaluate for inducible ventricular tachycardia that may benefit from primary prevention ICD implantation (Class IIB, Level of evidence C).^21^

CMR is a powerful tool that can provide comprehensive cardiac information to aid in the diagnosis of CS, including left ventricular systolic function, ventricular volumes and dimension, myocardial thinning, wall motion abnormalities, myocardial edema, and LGE. However, the most useful CMR parameter in the diagnosis of CS is the presence or absence of LGE. It should be borne in mind, that because LGE accumulation represents areas of underlying fibrosis, this can be a non-specific finding and can occur in various conditions, including scar from previous myocarditis or fibrosis from other cardiomyopathies. Although the typical LGE pattern in CS is midmyocardial and subepicardial involvement, occasionally CS may demonstrate subendocardial LGE, and thus mimic a prior myocardial infarction. Our analysis supports the clinical practice of using CMR to estimate the likelihood of CS and obtain prognostic information.

In patients with the suspicion of CS, the absence of LGE has a sufficiently high negative predictive value and reassuring event-free survival that no further testing would be required for the majority of patients. In our analysis, the majority of the patients (495/694 patients; 71%) did not have any evidence of LGE. This group of LGE-negative patients had low annualized incidence rates of cardiovascular mortality and ventricular arrhythmias at 0.6% and 0%, respectively. This confers a 68% and 100% relative risk reduction as compared with patients who were LGE positive, respectively. Similarly, for the combined outcome of mortality or ventricular arrhythmias, the annualized incidence rate was 10.8% versus 0.6% for LGE positive versus LGE negative, a 94% relative risk reduction. However, it should be noted that these risk differences according to LGE occur in the majority of studies that included sarcoidosis patients with known or suspected cardiac involvement. A relative outlier study with discordant findings by Nagai et al^10^ merits closer inspection. In the study by Nagai et al^10^, the population was explicitly a screening cohort that excluded any patients with potential cardiac symptoms and thus had the lowest prevalence of LGE+ scans among all 7 studies thereby reducing statistical power. Although the study by Patel et al^4^ did reach out to pulmonary and rheumatology clinics to recruit sarcoidosis patients, 21% of patients had possible cardiac symptoms. Furthermore, the use of corticosteroids was among the lowest in the study by Nagai et al^10^ (16%) when compared with the study by Patel et al^4^ (91%) and other studies, suggesting the patients in the study by Nagai et al^10^ had less active sarcoid and were a lower risk population. Additionally, the study by Nagai et al^10^ did not use 2 blinded experienced readers (clinical reports reviewed retrospectively), whereas 5 of 7 studies did report the interpretation by 2 blinded readers (Table 2), and the study by Nagai et al^10^ had other previously mentioned differences in scan acquisition. Whether this influenced the sensitivity of CMR to detect LGE is not known, but is possible, because the use of a fixed inversion time without nulling myocardium is not a standard practice. Consequently in the study by Nagai et al^10^, there were no arrhythmic events or cardiac deaths among the LGE-positive subjects and the only events were 3 all-cause deaths among the LGE-negative group. Thus, these outlying results underscore the importance of having a clinical suspicion of cardiac involvement of sarcoidosis before referring to CMR.

Despite the low event rates in patients without LGE on CMR, it is worth noting that cardiovascular death occurred in 2 patients without the evidence of LGE on CMR. Also, as mentioned earlier, because LGE accumulation represents underlying fibrosis, some patients referred for CMR with the suspicion of CS may have nonspecific LGE findings. In such cases, in patients with negative LGE by CMR but a high clinical suspicion, or in patients with pacemakers or ICDs who cannot undergo an MRI, cardiac PET may be useful for further evaluation.^20^ Another application of cardiac PET imaging in these patients is to assess the response to immunosuppressive therapy, particularly in light of recent data showing that a reduction in myocardial inflammation, as assessed by fluoro-deoxyglucose PET, is associated with an improvement in ejection fraction.^22^ Further studies are necessary to evaluate whether fluoro-deoxyglucose imaging provides incremental prognostic information among patients who are LGE positive.

While interpreting the results of our study, it is important to keep in mind that despite the higher event rate among patients with LGE, not all the patients with LGE develop adverse outcomes. To further stratify these patients and obtain additional prognostic information beyond the presence or absence of LGE, potentially significant clinical variables may include left ventricular ejection fraction, right ventricular ejection fraction, ECG abnormalities, race, and sex. Moreover, other information, which can be potentially obtained from routine CMR and utilized to risk-stratify these patients, could include the amount of LGE, T1 and T2 mapping, and an evaluation of left and right ventricular function. In this meta-analysis, we were unable to evaluate these variables, as these variables were not systematically reported in each study. Future longitudinal follow-up studies, ideally from multicenter registries, will be required to evaluate if these parameters can further risk-stratify both the LGE-positive and LGE-negative cohorts.

Our results are subject to limitations inherent to any meta-analysis based on pooling of data from different studies with different inclusion criteria, different designs, variable follow-up duration, and different patient populations. However, despite varying patient characteristics, our results did not demonstrate any significant statistical heterogeneity. Because we did not have access to raw data of individual patients, it was not possible to adjust for various patient factors, including age, sex, ejection fraction, race, prior or current use of steroids, and other medical therapies. Nevertheless, studies included in this analysis that were powered to adjust for these variables indicated that LGE was a more significant predictor of prognosis than left ventricular ejection fraction and these other covariates. Another important limitation is that most studies had relatively small sample sizes, as the majority of the studies are single-center studies. However, the results were consistent across diverse sites and the use of pooled data analysis provides statistical power that was lacking in several of the individual publications. Finally, because of the lack of events in some analyses, the relative risk estimates may be imprecise, which most significantly affects the outcome of ventricular arrhythmia because no events occurred among those without LGE. However, this would not affect the significant and clinically meaningful absolute differences in annualized event rates.

## Conclusions

The presence of LGE on cardiac MRI among patients with known or suspected CS provides strong prognostic information with regard to future all-cause mortality, cardiovascular mortality, and ventricular arrhythmia. Although the presence of LGE in 29% of patients was associated with a high rate of adverse events, the absence of any LGE offered the reassurance of a low-risk prognosis of ventricular arrhythmia or cardiovascular death.

## Supplementary Material

**Supplemental Table 2**. Quality Assessment Using Newcastle Ottawa Scale.

## Figures and Tables

**Table 1:** Baseline Demographic

Study	n	+ LGE, n (%)	% LGE Mass	Age, y	Men, n (%)	Ethnicity, %	Extra-Cardiac Sarcoid, n (%)	Follow-Up, mo	LVEF, %	LVEDV	MRI Field Strength, Tesla	Corticosteroid Treatment, n (%)*
Murtagh et al^5^	205	41 (20)	8±10	56±7	64 (31)	59 AA	205 (100)	36±18	61±6	73±15 mL/m^2^	1.5	92 (45) current
Patel et al^4^	81	21 (26)	6 (2, 19)	46±11	25 (31)	73 AA	81 (100)	21±8	56 (48, 61)	101 (89,137) mL	1.5	74 (91) current or prior
Greulich et al^6^	153	39 (25)	4 (3, 9)	50±13	91 (59)	…	153 (100)	31	63 (59, 68)	126 (105,155) mL	1.5	110 (72) current or prior
Nadel et al^8^	106	32 (30)	…	51±12	64 (60)	…	100 (94)	37±21	57±11	…	…	61 (58) current or prior
Crawford et al^9^	51	19 (37)	15±12	51±10	16 (32)	47 AA	51 (100)	48±20	52±10	175 mL	1.5	24 (47) current or prior
Nagai et al^10^	61	8 (13)	…	57±15	13 (21)	100 Japanese	61 (100)	50±12	63±7	105 mL	1.5	10 (16) current or prior
Shafee et al^7^	37	26 (70)	…	57±12	4 (12)	100 Japanese	30 (81)	45±31	50±16	LVEDd=49±8 mm	1.5	No steroids before CMR
Overall	694	199 (29)	…	53±4	277 (40)	…	681 (98)	36±8	59±4	…	1.5	…

Columns represent n(%) or mean±SD or median (IQR), where appropriate. AA indicates African American; LGE, late gadolinium enhancement; LVEDd, left ventricular end diastolic diameter; LVEDV, left ventricular end diastolic volume; LVEF, left ventricular ejection fraction; MRI, magnetic resonance imaging; and VT, ventricular tachycardia.

**Table 2:** Cardiac Magnetic Resonance Acquisition and Interpretation of Late Gadolinium Enhancement

Study	LGE Acquisition Time After Gadolinium, min	Gadolinium Type and Dose	Sequence	Interpretation of LGE Presence and Quantification
Murtagh et al^5^	10	Gadodiamide or gadobenate dimeglumine (0.1–0.2 mmol/kg)	T1-weighted GRE with PSIR (TI 200–300 ms)	>5 SDs above the mean signal intensity of normal remote myocardium, irrespective of LGE pattern or location. LGE quantified as a % of LV mass (%LGE)
Patel et al^4^	10	Gadoversetamide (0.15 mmol/kg)	Segmented inversion recovery GRE (TI 280–360 ms)	2 blinded readers using a semiquantitative 5-point LGE scale for each myocardial segment. LGE quantified by summing each segments' score and dividing by 17
Greulich et al^6^	5–10	Gadodiamide or Gadopentetate dimeglumine (0.15 mmol/kg)	Segmented inversion recovery fast GRE	2 blinded readers using the Siemens Argus analysis software package, and the results were expressed as percentage of myocardial mass
Nadel et al^8^	Not reported	Not reported	Not reported	2 blinded readers assessed LGE as typical for cardiac sarcoidosis when present in a nonvascular distribution in 2 orthogonal views and other potential clinical causes of LGE could be excluded
Nagai et al^10^	10	Gadopentetate dimeglumine (0.15 mmol/kg)	Inversion recovery SSFP (TI fixed 300 ms)	LGE determined by retrospective review of CMR reports
Crawford et al^9^	15	Gadopentetate dimeglumine (0.20 mmol/kg)	Inversion recovery GRE (TI 250–360 ms)	2 blinded readers rated LGE by visual scoring. LGE quantified as % of the left ventricular mass, using the Full-Width-Half-Maximum method
Shafee et al^7^	10–15	Gadopentetate dimeglumine (0.15 mmol/kg)	Inversion-recovery GRE (TI 200–300 ms)	2 experienced readers blinded to outcomes determined LGE+ or LGE−

Five studies reported the use of 2 experienced, blinded readers for CMR interpretation, whereas Nagai et al and Murtagh et al used retrospective review of the reports of previously clinically interpreted CMR. CMR indicates cardiac magnetic resonance; GRE, gradient recall echo; LGE, late gadolinium enhancement; and TI, inversion time used to null myocardium during LGE sequence.

**Table 3:** Definitions of Adverse Clinical Outcomes Reported by Each Study

Study	Arrhythmia End Points Reported	Clinical Outcomes
Murtagh et al^5^	Sustained ventricular arrhythmia (ie, lasting ≥30 s or any polymorphic VT), or appropriate ICD shock	Primary: All-cause mortality, sustained ventricular arrhythmia (ie, lasting ≥30 s or any polymorphic VT), or appropriate ICD shock
Patel et al^4^	Ventricular tachyarrhythmia leading to appropriate ICD discharge (based on stored electrograms) or symptomatic bradyarrhythmia leading to pacemaker implantation	Primary: Composite of all-cause mortality or symptomatic ventricular arrhythmia
Greulich et al^6^	Aborted SCD defined as resuscitation after cardiac arrest (cardioversion and/or cardiopulmonary resuscitation in a patient who remains alive 28 d later). Appropriate ICD shock defined as triggered by VT or VF and documented by stored intracardiac electrocardiographic data. VT defined as ≥3 ventricular beats >120 bpm for >30 s. NSVT defined as 3 or more ventricular beats >120 bpm for > 30 s	Primary: death, aborted sudden cardiac death, and appropriate ICD therapy Secondary: VT or NSVT
Nadel et al^8^	Sudden cardiac death, VT, VF, appropriate ICD therapy excluding antitachycardia pacing	Primary: Composite of SCD, VT, and VF Secondary: All-cause mortality, CV mortality, and VA (VT, VF, and appropriate ICD therapy excluding antitachycardia pacing)
Nagai et al^10^	Ventricular arrhythmia with clinical symptoms and necessitating admission or bradyarrhythmia leading to pacemaker implantation.	Primary: composite of all-cause death, heart failure admission, and symptomatic arrhythmia
Crawford et al^9^	Cardiac arrest or VT/VF on ECG or device electrogram lasting at least 30 s or requiring defibrillation.	Primary: ventricular arrhythmiaSecondary: All-cause mortality, CV mortality, VT or VF, and combined death or VT/VF
Shafee et al^7^	VA identified by 12-lead ECG, Inpatient telemetry or Holter ECG and defined as 3 or more consecutive beats of ventricular origin, at a rate of more than 100 bpm, thus including NSVT, VT, and VF	Primary: ventricular arrhythmia Secondary: composite of VA, heart failure hospitalizations, and cardiovascular mortality.

For meta-analysis, bradyarrhythmia and NSVT were excluded. CV indicates cardiovascular; ICD, implantable cardiac defibrillator; NSVT, nonsustained ventricular tachycardia; SCD, sudden cardiac death; VA, ventricular arrhythmia; VF, ventricular fibrillation; and VT, ventricular tachycardia.

**Table 4:** Adverse Clinical Outcomes According to Late Gadolinium Enhancement

			All-Cause Mortality		Ventricular Arrhythmia		Death or Ventricular Arrhythmia		CV Mortality		Pacemaker		Heart Failure Admission	
Study	n	Follow-Up, y	LGE Positive (n=173), n (%)	LGE Negative (n=484), n (%)	LGE Positive (n=199), n (%)	LGE Negative (n=495), n (%)	LGE Positive (n=199), n (%)	LGE Negative (n=495), n (%)	LGE Positive (n=132), n (%)	LGE Negative (n=288), n (%)	LGE Positive (n=59), n (%)	LGE Negative (n=159), n (%)	LGE Positive (n=40), n (%)	LGE Negative (n=127), n (%)
Murtagh et al^6^	205	3.0	6 (4.9)	2 (0.4)	4 (3.3)	0 (0)	10 (8.1)	2 (0.4)	…	…	…	…	…	…
Patel et al^5^	81	1.8	4 (10.5)	2 (1.8)	2 (5.2)	0 (0)	6 (15.7)	2 (1.8)	4 (10.5)	1 (0.9)	…	…	…	…
Greulich et al^6^	153	2.6	3 (3)	1 (0.3)	7 (6.9)	0 (0)	11 (10.8)	1 (0.3)	3 (3)	0 (0)	…	…	…	…
Nadel et al^8^	106	3.1	4 (4.1)	8 (3.5)	8 (8.2)	0 (0)	12 (12.2)	9 (4)	3 (3.1)	1 (0.4)	4 (0.6)	1 (0.1)	15 (2.4)	3 (0.2)
Nagai et al ^10^	61	4.2	0 (0)	3 (1.4)	0 (0)	0 (0)	0 (0)	3 (1.4)	0 (0)	0 (0)	1 (0.1)	0 (0)	0 (0)	0 (0)
Crawford et al^9^	51	4.0	2 (1.6)	1 (1.3)	13 (10.2)	0 (0)	14 (10.9)	1 (1.3)	1*(0.8)	…	1 (0.1)	1 (0.1)	…	…
Shafee et al^7^	37	3.8	…	…	7 (7.2)	0 (0)	11 (11.3)	0 (0)	…	…	…	…	…	…
Pooled	694	…	19 (3.2)	17 (0.6)	41 (5.9)	0 (0)	64 (10.8)	18 (0.6)	10 (1.9)	2 (0.6)	…	…	…	…

Columns represent absolute (% annualized incidence). Outcomes pooled by random effects meta-analysis are reported in the final row. Only 3 studies reported 6 pacemaker implantations because of heart block, and 2 studies reported heart failure exacerbations; these 2 outcomes with limited data were not pooled.

* CV mortality from Crawford^9^ was not included in pooled outcome because of unknown cause of death in comparison group. CV indicates cardiovascular; and LGE, late gadolinium enhancement.

## Clinical Perspective

Myocardial infiltration occurs in ≈25% of patients with known extra-cardiac sarcoidosis and is increasingly recognized as an important cause of arrhythmia and death. Multiple clinical criteria have been proposed to aid in diagnostic and prognostic evaluation of known or suspected cardiac sarcoidosis, starting with the 1993 (revised in 2007) Japanese Ministry of Health and Welfare criteria to, more recently, an expert consensus statement proposed by the Heart Rhythm Society. However, such clinical criteria lack sensitivity, and the evaluation for late gadolinium enhancement (LGE) by cardiac magnetic resonance has been demonstrated in several small cohorts to have a potential prognostic role. However, these have been limited by small sample size leading to conflicting results. In our systematic review and meta-analysis, we evaluated the prognostic value of cardiac magnetic resonance for patients with known or suspected cardiac sarcoidosis. Among 7 studies of 694 patients, 29% were LGE positive. We demonstrated a markedly increased risk of all-cause mortality, ventricular arrhythmia, cardiovascular mortality, and combined death or ventricular arrhythmia. These findings bear significance because consensus opinions for the diagnosis of cardiac sarcoidosis have not been validated scientifically. In the limited studies comparing such clinical criteria to LGE by cardiac magnetic resonance, the LGE findings offer superior diagnostic and prognostic value. Thus, abnormal LGE findings may be used to counsel nearly one third (29% LGE+ in our analysis) of sarcoidosis patients of increased risk of future death or ventricular arrhythmia. Furthermore, a normal cardiac magnetic resonance conveys a powerful, low-risk prognosis among the patients in these cohorts.
